# The Localization Research of Brain Plasticity Changes after Brachial Plexus Pain: Sensory Regions or Cognitive Regions?

**DOI:** 10.1155/2019/7381609

**Published:** 2019-01-08

**Authors:** Shuai Wang, Zhen-zhen Ma, Ye-chen Lu, Jia-jia Wu, Xu-yun Hua, Mou-xiong Zheng, Jian-guang Xu

**Affiliations:** ^1^School of Rehabilitation Science, Shanghai University of Traditional Chinese Medicine, Shanghai, China; ^2^Key Laboratory of Hand Reconstruction, Ministry of Health, Shanghai, China; ^3^Shanghai Key Laboratory of Peripheral Nerve and Microsurgery, Shanghai, China; ^4^Department of Rehabilitation, Yueyang Hospital of Integrated Traditional Chinese and Western Medicine, Shanghai University of Traditional Chinese Medicine, Shanghai, China; ^5^Department of Trauma and Orthopedics, Yueyang Hospital of Integrated Traditional Chinese and Western Medicine, Shanghai University of Traditional Chinese Medicine, Shanghai, China; ^6^Department of Hand Surgery, Huashan Hospital, Fudan University, Shanghai, China

## Abstract

**Objective:**

Neuropathic pain after brachial plexus injury remains an increasingly prevalent and intractable disease due to inadequacy of satisfactory treatment strategies. A detailed mapping of cortical regions concerning the brain plasticity was the first step of therapeutic intervention. However, the specific mapping research of brachial plexus pain was limited. We aimed to provide some localization information about the brain plasticity changes after brachial plexus pain in this preliminary study.

**Methods:**

24 Sprague-Dawley rats received complete brachial plexus avulsion with neuropathic pain on the right forelimb successfully. Through functional imaging of both resting-state and block-design studies, we compared the amplitude of low-frequency fluctuations (ALFF) of premodeling and postmodeling groups and the changes of brain activation when applying sensory stimulation.

**Results:**

The postmodeling group showed significant decreases on the mechanical withdrawal threshold (MWT) in the bilateral hindpaws and thermal withdrawal latency (TWL) in the left hindpaw than the premodeling group (*P* < 0.05). The amplitude of low-frequency fluctuations (ALFF) of the postmodeling group manifested increases in regions of the left anterodorsal hippocampus, left mesencephalic region, left dorsal midline thalamus, and so on. Decreased ALFF was observed in the bilateral entorhinal cortex compared to that of the premodeling group. The results of block-design scan showed significant differences in regions including the limbic/paralimbic system and somatosensory cortex.

**Conclusion:**

We concluded that the entorhinal-hippocampus pathway, which was part of the Papez circuit, was involved in the functional integrated areas of brachial plexus pain processing. The regions in the “pain matrix” showed expected activation when applying instant nociceptive stimulus but remained silent in the resting status. This research confirmed the involvement of cognitive function, which brought novel information to the potential new therapy for brachial plexus pain.

## 1. Introduction

Neuropathic pain, which is caused by a lesion or disease affecting the somatosensory nerve system [1], remains an increasingly prevalent and intractable disease due to inadequacy of satisfactory treatment strategies [2]. Brachial plexus avulsion (BPA) is one of the most severe peripheral nerve injuries, which affect the function of the upper extremity. Apart from the motor and sensory deficit, brachial plexus pain was described as stimulus-induced, spontaneous ongoing, and burning. Also, this kind of pain was reported to be resistant to most traditional pain relief treatments [3]. According to the epidemic researches, the incidence of neuropathic pain after brachial injury has reached 50% [3–6]. Considering its severe syndrome and high occurrence, it becomes a vital problem in the clinical practice and is worth deep mining.

Previous studies have proved that plastic changes of various brain regions concerning pain processing contributed to the recovery of neuropathic pain. Those regions mainly involved the prefrontal-limbic-brainstem areas, the somatosensory cortices, the prefrontal cortex (PC) [7], the insular cortex (IC), the anterior cingulate cortex (ACC) [8], the basal ganglion regions, the hypothalamus (HT), and the ventral midbrain (VMB) [9, 10]. All of these changes were considered a maladaptive response of noxious damage by increased activation and reduced functional connectivity of subcortical pathways, which were believed to contribute to the increased pain sensitivity [11, 12]. The modern neuroscience held the view that cortical reorganization was a significant indicator in the prognosis of sensorimotor function following nerve injury and repair [13–18].

As central plasticity is involved in the long-term sensitivity changes in neuropathic pain [12], it is noteworthy to determine how the brain plasticity evolves in the occurrence and development of pain after BPA. However, the localization neuroimaging study about brachial plexus pain is very limited and blurred [19, 20]. Most people held the view that neuropathic pain was just an issue related to the somatosensory cortex. Through our present study, we would map the associated regions under both task-dependent and task-independent circumstances on a highly controlled animal model. Thus, we could provide some reliable and novel information about cortical plasticity of neuropathic pain after BPA, which would be useful in the discovery of new treatment for pain relief.

## 2. Materials and Methods

### 2.1. Animals

Experiments were conducted on 50 adult female Sprague-Dawley (SD) rats (average weight 200–250 g). Shanghai Slack Laboratory Animal Limited Liability Company (Shanghai, China) provided all of the rats, which lived under the conditions with 12-hour light/dark cycle and enough food or water. Before further intervention or examination, the rats were kept at least 7 days. After model establishment, we screened the rats based on the presence of self-harm behavior in order to distinguish which of them presented neuropathic pain syndrome. 24 out of 50 remained in the study after the selection.

All protocols and procedures of animals followed the guidelines of the Biomedical Resource Center and were approved by the Institutional Animal Care and Use Committee.

### 2.2. Establishment of Animal Models

The rats received intraperitoneal injection of anesthesia by sodium pentobarbital (40 mg/kg) and then were placed lying prostrate on a clean surgical table. We aimed to establish the models by damaging the right brachial plexus. A skin incision of approximately 4 cm in length was made through the dorsal midline, and the paraspinal muscles were separated with a pair of ophthalmic scissors. Using the prominent spinous process of the C7 vertebrae as a landmark, the hemilaminectomies from C4 to T1 on the right were performed under the operating microscope (magnification ×10) to expose the spinal cord through a posterior surgical approach. Once the whole roots from C5 to T1 had been clearly confirmed, they were avulsed from the spinal cord with a micronerve hook. Bipolar electrocoagulation was applied to promote hemostasis during operation. And then, the wound was closed in layer with penicillin powder. Postoperatively, rats were placed on a heated blanket to recover for 1~2 hours and then returned to their cages. All surgeries were performed by the same experimenter full of experience.

### 2.3. Behavioral Assessment (Mechanical Withdrawal Threshold and Thermal Withdrawal Latency)

Withdrawal responses to mechanical and thermal stimuli were measured at the time of premodeling and 1-month postmodeling. Prior to behavioral testing, rats were habituated to the room for 1 hour in their home cages followed by a 30-minute habituation to the testing apparatus.

Mechanical sensitivity was measured on both hindpaws using a series of ascending force von Frey monofilaments (Stoelting, IL). Animals were placed on a metal mesh floor, covered by a transparent plastic box and raised 30 cm above the floor. The threshold was taken as the lowest force that evoked a brisk withdrawal response, at least two out of four times repetitive stimuli. A withdrawal response was considered valid only when the paw was completely removed from the platform [21].

We used the Plantar Test Apparatus (Hargreaves method) for mice and rats (Chengdu Techman Software Co. Ltd., Chengdu, China) to evaluate the extent of thermal withdrawal latency. The rats were put on an elevated glass floor platform and underside a high-intensity moveable radiant heat source. Then, we moved its alignment to the lateral plantar surface of the rat's hindpaws. The withdrawal latency was measured from the onset of heat until the hindpaws moving back, as shorter latency suggested more pain. Each hindpaw was estimated three times and at a five-minute interval. The maximal automatic cutoff latency to avoid tissue damage was reached in 20 sec [22]. In addition, we select the bilateral hindpaws as targets because they appeared to be better indicators of overall pain threshold. Although the hindpaws were unaffected, the pain threshold of hindpaws would be influenced by the neuropathic pain aroused by brachial plexus avulsion of frontpaws. In other words, the injury caused peripheral sensitization of nociceptors [23]. All the procedures were in line with our prior work [24], which ensured homogeneity in the series studies.

### 2.4. fMRI Data Acquisition

All fMRI scans were performed on a 7 T horizontal-bore Bruker scanner (Bruker Corporation, Germany), which was equipped with a gradient system of 116 mm inner diameter and a maximum gradient strength of 400 mT/m. The fMRI scan was carried out premodeling and 1 month after modeling. The sequence of fMRI scan consists of one session of resting state and two sessions of block design. Rats were anesthetized with 4% isoflurane at the beginning and then fixed on the scanner and maintained with 1.5%–2% isoflurane in oxygen-enriched air (20% oxygen/80% air) with necessary ventilator support. This research utilized a single transmit and receive surface coil consisting of a single copper wire loop.

An interleaved single-shot EPI sequence was applied for functional imaging of both task state and resting state. Parameters are listed as follows: flip angle = 90°, slice thickness = 0.5 mm, repetition time = 3000 ms, echo time = 20 ms, number of averages = 1, and FOV = 32^∗^32 mm with 64^∗^64 points. EPI fMRI volumes covered a relatively restricted area which was approximately centered on the bregma point. In order to minimize the confounders in the animal model data set, we adopted the same scanning parameters of BOLD as our prior work [24].

For functional imaging of the task state, the whole scan began with a dummy epoch of 8 seconds, which would be automatically discarded by the system. Both “ON” and “OFF” epochs lasted for 30 seconds, and these two epochs sequentially formed one cycle. We designed 10 cycles totally in one stimulation session, during which only one side forelimb was stimulated with electric needles inserted beneath the skin of each forelimb. Two needle electrodes were set in the proximal and distal ends of the forelimb, respectively. In order to avoid habituation of sensory stimulation, the stimulus was performed in a pseudorandom pattern.

### 2.5. fMRI Data Processing

The process of data processing and analysis was performed by the Statistical Parametric Mapping 8 toolbox (http://www.fil.ion.ucl.ac.uk/spm/) on a MATLAB 2014a platform. At the first part of processing, images were enlarged ten times and turned to a human brain size approximately, which enabled it possible to use the processing algorithms originally developed for human data [25, 26].The upscaling procedure only changed the dimension descriptor fields in the file header, and no interpolation was applied. The nonbrain tissue was stripped manually before statistical analysis. The fMRI images were firstly corrected from the temporal bias of slice acquisition by a slice timing procedure. The images were spatially realigned with rigid-body transformations in order to account for voxel misplacement caused by in-scanner head motion. The transformation matrix was estimated by the mean image and a standard template. Then, the matrix was written to functional images for individual MNI normalization. In the final phase, the images were smoothed by a FWHM (full width at half maximum) twice as the voxel size.

Task-state data were statistically analyzed using SPM8. A mass-univariate approach based on general linear models (GLMs) was conducted for the statistical analysis of fMRI data. Then, we determined a GLM design matrix according to the experimental paradigm and estimated the GLM parameters through classical or Bayesian approaches in SPM. The results were interrogated using contrast vectors to produce statistical parametric maps (T maps in this study). The threshold was set at *P* < 0.05 with FDR (false discovery rate) correction.

Analyses for resting-state data were conducted by using REST (Beijing Normal University, http://www.restfmri.net) to calculate ALFF data. The linear detrending and band-pass filtering (0.01–0.08 Hz) were performed on the resting-state time series, followed by regressing out the mean time series of global, white matter, and cerebrospinal fluid signals, to remove artifacts and reduce physiological noise. For the ALFF analysis, the time series of each voxel was transformed to the frequency domain using fast Fourier transform. The power spectrum was acquired in the frequency band at each voxel. As the power of a given frequency was proportional to the square of the amplitude of this frequency component, the square root of the power spectrum was calculated at each frequency. Then, the averaged square root (i.e., ALFF value) of the power spectrum in different frequency bands was calculated [27]. Finally, each individual's ALFF value was transformed to *Z* score to make further comparison between groups.

### 2.6. Statistical Analysis

The significant activation area and ALFF value within groups through one-sample *t*-test were reported and then binarized to a mask (value one within the mask and zero out of the mask). Paired-sample *t*-test was adopted to identify the difference of sensory stimulus response and ALFF value between pre- and postmodeling. The statistical comparison was carried out within the boundary of a previously generated mask. Thresholds were set at *P* < 0.05 with FDR (false discovery rate) correction. SPSS21.0 statistical software was used to analyze the behavioral data. The data were shown as mean ± standard deviation. Values of *P* < 0.05 were considered statistically significant.

## 3. Results

### 3.1. Group Differences of Behavior Assessment

**Figure 1:**
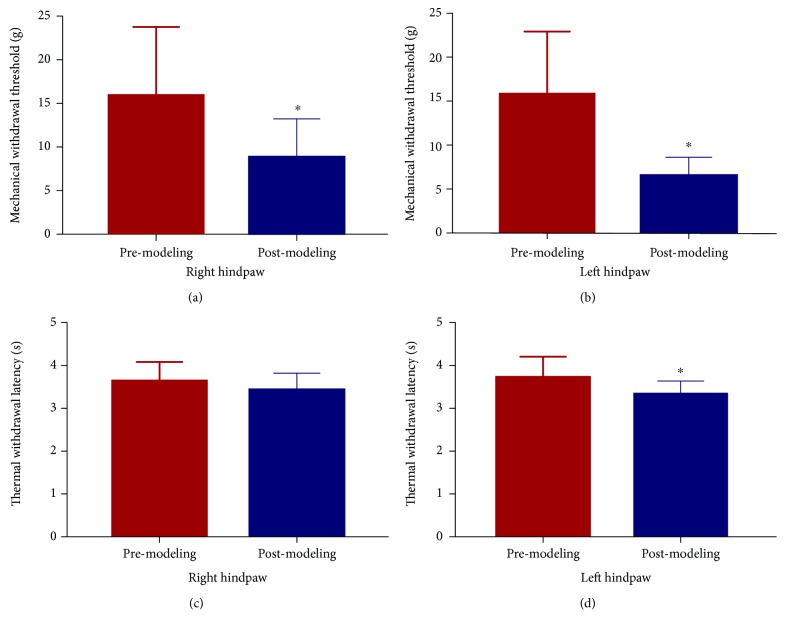
Results of mechanical withdrawal threshold (MWT) and thermal withdrawal latency (TWL). After modeling, there was a significant reduction of MWT on the right (a) hindpaw and the left (b) hindpaw. No significant difference was found between premodeling and postmodeling in TWL of the right hindpaw (c). Compared with premodeling, TWL of the left hindpaw (d) was significantly reduced postmodeling (^∗^ *P* < 0.01, compared with premodeling).

The assessment of bilateral hindpaws showed that there was a significant reduction of mechanical allodynia on the bilateral hindpaw and a significant reduction of thermal withdrawal latency on the left hindpaw after modeling. The thermal withdrawal latency of the right hindpaw showed no significant but trending reduction. Overall, the neuropathic pain caused a decrease in the MWT and TWL (see Figure 1).

### 3.2. Group Differences of ALFF Analysis in Resting-State Scan

**Figure 2:**
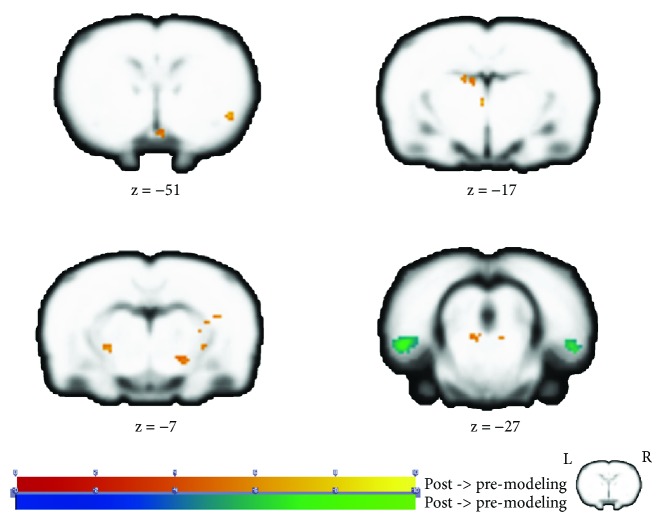
Group differences of ALFF analysis in resting-state scan. The warm tone represents that the ALFF value of postmodeling was higher than that of premodeling, while the cold tone represents that the ALFF value of postmodeling was lower than that of premodeling. *Z*-value was the *z*-axis coordinate along the anterior-posterior axis referenced to a stereotaxic rat brain MRI template [69] which has been aligned with the coordinates of Paxinos and Watson's. ALFF: amplitude of low-frequency fluctuations.

The statistical analysis showed increased ALFF in multiple cerebral regions, including the left anterodorsal hippocampus (ADH), left mesencephalic region, left dorsal midline thalamus (DMT), right mesencephalic region, right dorsolateral thalamus (DLA), and right internal capsule (ic) as well as the right piriform cortex (Pir) after modeling. Additionally, we found decreased ALFF values in the bilateral entorhinal cortex (EC) (see Figure 2 and Table 1).

### 3.3. Group Differences of Activation in Block-Design Scan

**Figure 3:**
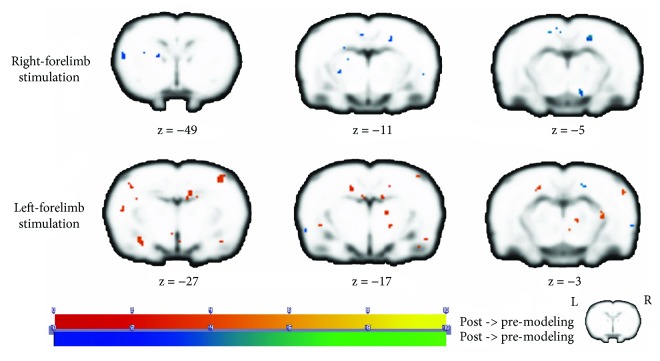
Group differences of activation in block-design scan. The first row in the figure displays results of the right forelimb stimulation task, and the second row displays results of the left forelimb stimulation task. The warm tone represents activation of postmodeling greater than premodeling, whereas the cold tone represents activation of postmodeling weaker than premodeling. *Z*-value was the *z*-axis coordinate along the anterior-posterior axis referenced to a stereotaxic rat brain MRI template [69] which has been aligned with the coordinates of Paxinos and Watson's.

From the analysis of right limb stimulation task, the statistical analysis showed that no increased activation was found after remodeling. We observed decreased activation in the left piriform cortex (Pir), left orbitofrontal cortex (OFC), left retrosplenial cortex (RSC), left medial prefrontal cortex (mPFC), right posterior hippocampus (pHPC), and right piriform cortex (Pir) (see Figure 3 and Table 2).

From the analysis of left limb stimulation task, the statistical analysis showed increased activation in the left somatosensory cortex (SmI), left caudate putamen (CPu), left dorsolateral thalamus (DLA), right orbitofrontal cortex (OFC), and left piriform cortex (Pir) after remodeling. We observed decreased activation in the right posterodorsal hippocampus (PDH), right retrosplenial cortex (RSC), right lateral hypothalamus (LH), right ventral hippocampus (vHIP), bilateral amygdala (AMY), and right AcbC (accumbens nucleus, core) (see Figure 3 and Table 3).

## 4. Discussion

Brachial plexus pain, characterized by allodynia (painful perception of innocuous stimuli), hyperalgesia (increased sensitivity to painful stimuli), and spontaneous pain, is still a big challenge in the clinical practice. Consequently, researchers are often disappointed by the poor pain relief outcome of various physical therapies for BPA patients. Current opinions on the central mechanism of neuropathic pain suggested a series of brain regions, which presented a fixed pattern of activation and were together named as “pain matrix” by Ingvar [28–30]. The studies revealed that the neuropathic pain was not only an issue concerning somatosensory areas but also cognition-related [31–34]. However, there was no research which specifically focuses on the localization of activated brain regions of brachial plexus pain. The lack of spatial information resulted in a lot of blindly designed stimulation therapies and failed to achieve any pain relief. The current study provided a controlled animal model and investigated the longitudinal functional changes of the brain under both electric stimulation and resting state.

From the analysis of resting-state data, we found that there were significant differences of ALFF values in brain regions including the hippocampus and entorhinal cortex. Specifically, regions of the thalamus, hippocampus, mesencephalon, and internal capsule as well as the piriform cortex showed significantly increased ALFF after modeling. Regions of the entorhinal cortex displayed decreased ALFF after modeling. Deafferentation disease was characterized as deficits of information input and then caused temporary or permanent silence of corresponding brain regions [35]. Previous studies on neuropathic pain described that the major change in the primary somatosensory cortex (S1) rewired synaptic connections [36]. Based on former findings, there were supposed to be alterations in the somatosensory cortex aroused by pain in this research. However, we surprisingly discovered no significant clusters in the somatosensory cortex based on the analysis of resting-state data, which implied no changes of spontaneous neural activity. Instead, brain regions concerning with cognitive processing and memory such as the hippocampus and entorhinal cortex were greatly influenced. Therefore, we hypothesized that the sustained pain of BPA was more associated with cognitive processing rather than isolated sensory processing.

Previous studies confirmed an important role of the hippocampus in pain processing [37–40]. The hippocampus acted as the processer of nociceptive information in the deleterious effects of chronic pain on cognitive, emotional, and motivational functions [41, 42]. Wei et al. [39] proved that the magnitude of EPSPs in hippocampal CA1 pyramidal cells was related to the intensity of nociceptive stimulation positively. These stimulation and lesion studies demonstrated that pain processing was a primary function of the hippocampus. This was consistent with our results of fMRI data analysis, which brought more supportive information to this topic.

Early findings suggested that the entorhinal cortex may be a source of pain modulation in the hippocampal formation [43]. The hippocampus and connected networks are integral parts of the Papez circuit and critical structures for learning, memory, and pain perception and processing [44–46]. The hippocampus belongs to the limbic system and plays an important role in the consolidation of information about emotion and memory, through subregion functional connections and/or participating in the Papez circuit. In 1937, James Papez proposed that the neural circuit connecting the hypothalamus to the limbic lobe was the basis for emotional experiences [47]. Recent studies showed that the Papez circuit had a more significant role in memory functions. The structure begins and ends with the hippocampus. It circuits the pathway, including the hippocampus subiculum, fornix, mammillary bodies, mammillothalamic tract, anterior thalamic nucleus, cingulum, and entorhinal cortex, and finally comes back to hippocampus formation [48]. It was initially believed that the circuit was involved with emotion [49] and memory [45, 50, 51]. Despite its physiological sense, some researches on clinical pathologies, such as Alzheimer's disease [52, 53] and mild cognitive impairment [54], indicated its key predictor role in advanced cognitive function in brain regions.

Our data suggested that the entorhinal cortex had a significant difference in task-independent circumstance. It implicated that the persistent peripheral pain processing of brachial plexus pain was closely associated with the entorhinal-hippocampus pathway. We believed that the increased spontaneous neural activity of the entorhinal-hippocampus pathway was a consolidation of pain-aroused plasticity even though the original trigger region was missing. From this angle, brachial plexus pain was more like a cognitive impairment than a sensory disorder. Considering the involvement of the hippocampus and entorhinal cortex in pain processing [55, 56], our findings may lead to new methods of pain relief with cognitive intervention.

In addition to the resting-state analysis, we also performed sensory stimulation task in block-design BOLD scan. The results demonstrated that brain regions including the basal ganglia and hippocampus were also involved. Among those located regions, IC, SmI, AMY, and thalamus were in line with the classic conception of pain processing areas, which were called “pain matrix” [57]. Each of them encodes specialized subfunctions such as sensory-discriminative, affective nodes [58, 59]. The magnitude of activity in this network was said to be robustly correlated with the intensity of perceived pain [60, 61]. We noticed that the sensory stimulation task aroused a neural activation pattern in line with classic pain processing. That differed a lot with the ALFF results revealed by resting-state analysis. We reasoned that “pain matrix” regions were called by the instant electric stimulus to process the nociceptive information. But the self-aware pain of brachial plexus was more like a cognitive status, which activated the entorhinal-hippocampus Papez circuit in the resting state. Previous studies reported selectively modulated the sensory and affective dimensions of pain, using a cognitive intervention, which would promote handling these psychological dimensions of pain and changes in physiological responses to the noxious stimuli [62–65]. Therefore, we could infer that brachial plexus pain was a complex combination of sensory and cognitive plasticity, which probably resulted in refractory pain of BPA patients.

In addition, the block-design analysis revealed other multiple regions of altered activation. The thalamus is well believed to act as a relay station, delivering information between subcortical areas and the cerebral cortex. The thalamus has many connections to the hippocampus which may contribute to the Papez circuit via the mammillothalamic tract, comprising the mammillary bodies and fornix [66]. Considering the role of the thalamus as a relay station, we believed that the variation of activation in such regions was a result of altered efferentation from higher-level regions. The orbitofrontal cortex (OFC) is a prefrontal cortex region which has been proposed to be involved in sensory integration, in representing the affective value of reinforces, decision-making, and expectation. The caudal OFC is heavily interconnected with sensory regions, notably receiving direct input from the piriform cortex and the amygdala [67]. In human fMRI studies, the retrosplenial cortex (RSC) has emerged as a key number of a core network of brain regions that underpins a wide range of cognitive functions, including episodic memory, navigation, imaging future events, and processing scenes. Both cognition-related areas such as OFC and RSC were reported to be compromised in the most common memory-related neurological disorders [68], which was in line with our theory for brachial plexus pain.

## 5. Conclusions

We concluded that the functional integrated areas of brachial plexus pain processing involved the entorhinal-hippocampus pathway, which was part of the Papez circuit. The regions of “pain matrix” were in charge of the instant nociceptive stimulus but absent in the resting state. This research confirmed the involvement of cognitive function in the plasticity changes after brachial plexus pain, which might lead to a new therapeutic method for brachial plexus pain.

## 6. Limitation

This study utilized rat models under anesthesia condition, which could bring in unspecific activation and lower the significance of the result. In particular, we cannot explain the absence of the pain matrix element and the activation or altered spontaneous neural activity in brain regions such as the piriform cortex. More experiment and methodology should be applied for determination.

## Figures and Tables

**Table 1:** Differences in the ALFF between premodeling and postmodeling groups.

Brain regions	Extent	Cluster centroid (MNI)			*t*-value
		*x*	*y*	*z*	
*Post > Pre*					
L_Hippocampus_Antero_Dorsal	35	−15.29	5.05	−16.99	4.78
L_Mesencephalic_Region	21	−9.15	−17.69	26.97	4.47
L_Thalamus_Midline_Dorsal	46	−7.08	−11.47	−1.02	5.13
L_Thalamus_Midline_Dorsal	35	−2.96	−11.45	−17.02	6.11
R_Mesencephalic_Region	21	11.45	−19.78	28.97	4.67
R_Thalamus_Dorsolateral	21	30.02	−1.21	−13.00	5.17
R_ic	23	38.23	−17.71	−9.03	5.61
R_Cortex_Piriform	39	52.64	−25.90	−51.04	6.09
*Post < Pre*					
R_Cortex_Entorhinal	285	62.94	−28.10	24.96	−5.46
L_Cortex_Entorhinal	954	−58.60	−23.79	26.96	−6.01

*x*, *y*, and *z*: coordinates of primary peak locations in the MNI space; *t*-value: peak value of the cluster. FDR correction, *P* < 0.05.

**Table 2:** Differences of activation between premodeling and postmodeling when applying sensory stimulation task to the right forelimb.

Brain regions	Extent	Cluster centroid (MNI)			*t*-value
		*x*	*y*	*z*	
*Post > Pre*					
None					
*Post < Pre*					
L_Cortex_Piriform	85	−42	−26	−63	−4.678
L_Cortex_Orbitofrontal	83	−28	5	−87	−3.603
L_Cortex_Retrosplenial	124	−17	24	15	−6.041
L_Cortex_Medial_Prefrontal	64	−9	15	−83	−4.289
R_Cortex_Medial_Prefrontal	86	5	20	−91	−5.394
R_Hippocampus_Posterior	41	40	5	15	−4.675
R_Cortex_Piriform	27	53	−38	−37	−4.875

*x*, *y*, and *z*: coordinates of primary peak locations in the MNI space; *t*-value: peak value of the cluster. FDR correction, *P* < 0.05.

**Table 3:** Differences of activation between premodeling and postmodeling when applying sensory stimulation task to the left forelimb.

Brain regions	Extent	Cluster centroid (MNI)			*t*-value
		*x*	*y*	*z*	
*Post > Pre*					
L_Cortex_Somatosensory	48	−54	3	−73	−4.061
L_Cortex_Somatosensory	23	−61	−3	−49	−3.605
L_Caudate_Putamen	200	−36	−28	−59	−5.439
L_Caudate_Putamen	22	−21	−3	−51	−4.092
L_Thalamus_Dorsolateral	40	−32	−13	−9	−3.613
L_Thalamus_Dorsolateral	21	−21	−1	−11	−3.263
R_Cortex_Orbitofrontal	52	3	−3	−87	4.155
L_Cortex_Piriform	46	−28	−15	−85	3.606
*Post < Pre*					
R_Hippocampus_Postero_Dorsal	158	24	24	−7	−4.982
R_Cortex_Retrosplenial	24	16	23	15	−4.965
R_Hypothalamus_Lateral	40	13	−34	−5	−4.175
R_Hippocampus_Ventral	143	53	−16	1	−4.162
R_Amygdala	143	55	−28	−23	−4.036
R_Amygdala	49	53	−34	−15	−3.680
L_Amygdala	43	−54	−28	−25	−3.515
R_AcbC	43	18	−20	−65	−3.374

*x*, *y*, and *z*: coordinates of primary peak locations in the MNI space; *t*-value: peak value of the cluster. FDR correction, *P* < 0.05.

## Data Availability

The data used to support the findings of this study are available from the corresponding author upon request.

## Supplementary Materials

Supplementary Materials The hippocampus and entorhinal cortex pathway were discussed in this article. Resting-state fMRI analysis revealed a relatively larger extent of an ALFF significant area in the entorhinal cortex. It remained a question why the entorhinal cortex was activated while the hippocampus was not. We calculated the functional connectivity between the peak points of each region. The results showed that the functional connectivity did not change significantly after modeling. The two regions, which were involved in the Papez circuit, might play a different role in the brachial plexus pain processing.
