# Supplementary material for: The Localization Research of Brain Plasticity Changes after Brachial Plexus Pain: Sensory Regions or Cognitive Regions?
Source: Neural Plast. 2019 Jan 8;2019:7381609. doi: 10.1155/2019/7381609 (PMC6341257; doi:10.1155/2019/7381609)
Supplement: Supplementary Materials — The hippocampus and entorhinal cortex pathway were discussed in this article. Resting-state fMRI analysis revealed a relatively larger extent of an ALFF significant area in the entorhinal cortex. It remained a question why the entorhinal cortex was activated while the hippocampus was not. We calculated the functional connectivity between the peak points of each region. The results showed that the functional connectivity did not change significantly after modeling. The two regions, which were involved in the Papez circuit, might play a different role in the brachial plexus pain processing. [file 7381609.f1.pdf]

Supplementary Material

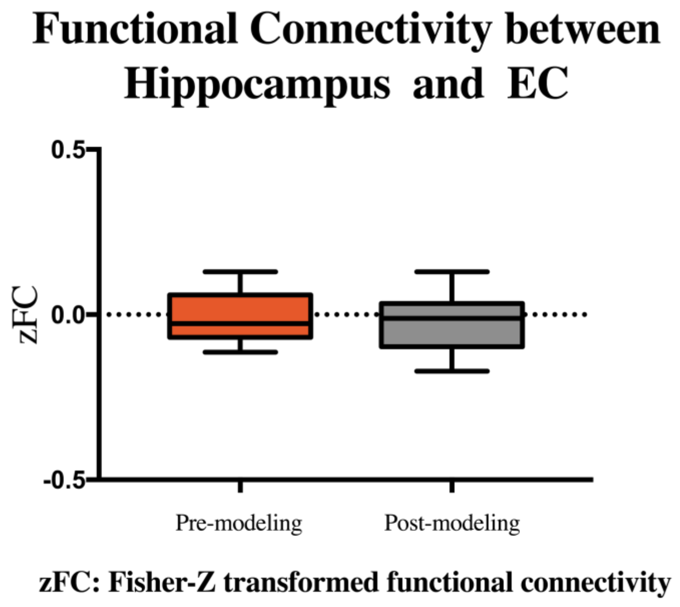

Figure Description: The hippocampus and entorhinal cortex pathway were discussed in this article. Resting-state fMRI analysis revealed a relatively larger extent of ALFF significant area in entorhinal cortex. It remained a question why the entorhinal cortex was activated meanwhile the hippocampus was not. We calculated the functional connectivity between the peak point of each region. The results showed the functional connectivity did not change significantly after modeling. The two regions, which were involved in the Papez Circuit, might play different role in the brachial plexus pain processing.
